# Intra-individual correlations between quantitative THK-5351 PET and MRI-derived cortical volume in Alzheimer’s disease differ according to disease severity and amyloid positivity

**DOI:** 10.1371/journal.pone.0226265

**Published:** 2019-12-13

**Authors:** Ji Eun Park, Jessica Yun, Sang Joon Kim, Woo Hyun Shim, Jungsu S. Oh, Minyoung Oh, Jee Hoon Roh, Sang Won Seo, Seung Jun Oh, Jae Seung Kim

**Affiliations:** 1 Department of Radiology and Research Institute of Radiology, University of Ulsan College of Medicine, Asan Medical Center, Seoul, South Korea; 2 Department of Nuclear Medicine, Asan Medical Center, University of Ulsan College of Medicine, Seoul, South Korea; 3 Department of Neurology, University of Ulsan College of Medicine, Asan Medical Center, Seoul, South Korea; 4 Department of Neurology, Samsung Medical Center, Sungkyunkwan University School of Medicine, Irwon-ro, Kangnam-ku, Seoul, South Korea; Nathan S Kline Institute, UNITED STATES

## Abstract

**Purpose:**

To assess the *in vivo* whole-brain relationship between uptake of [^18^F]THK-5351 on PET and cortical atrophy on structural MRI according to the presence and severity of Alzheimer’s disease (AD).

**Materials and methods:**

Sixty-five participants (21 normal controls, 32 mild cognitive impairment [MCI] subjects, and 12 AD patients) were enrolled from a prospective multicenter clinical trial (NCT02656498). Structural MRI and [^18^F]THK-5351 PET were performed within a 2-month interval. Cortical volume and standardized uptake value ratios (SUVR) were calculated from MRI and PET images, respectively, for 35 FreeSurfer-derived cortical regions. Pearson’s correlation coefficients between SUVR and cortical volume were calculated for the same regions, and correlated regions were compared according to disease severity and β-amyloid PET positivity.

**Results:**

No significantly correlated regions were found in the normal controls. Negative correlations between SUVR and cortical volume were found in the MCI and AD groups, mainly in limbic locations in MCI and isocortical locations in AD. The AD group exhibited stronger correlations (*r* = −0.576–0.781) than the MCI group (*r* = 0.368–0.571). Hippocampal atrophy did not show any correlation with SUVR in the β-amyloid PET-negative group, but negatively correlated with SUVR (r = −0.494, *P* = .012) in the β-amyloid PET-positive group.

**Conclusions:**

Regional THK-5351 uptake correlated more strongly with cortical atrophy in AD compared with MCI, thereby demonstrating a close relationship between the neuro-pathologic process and cortical atrophy. Hippocampal atrophy was associated with both β-amyloid and THK-5351 uptake, possibly reflecting an interaction between β-amyloid and tau deposition in the neurodegeneration process.

## Introduction

Ever since Braak and Braak demonstrated the neuropathological staging of Alzheimer’s disease (AD) with tau aggregates [1, 2], the noninvasive measurement of tau propagation has been of great clinical interest. The topographical distribution of tau aggregates on tau PET imaging has been described, with the tau burden first appearing in the transentorhinal region and then in the medial temporal lobes, and subsequently spreading to neocortical association areas [3, 4]. The progression of tau aggregates observed in an autopsy study seems to match the patterns of cortical atrophy on MRI [5], with a high tau burden being associated with greater cortical loss in medial and lateral temporal lobes. Noninvasive evaluation of the correlation between cortical atrophy and tau burden may advance our understanding of the pathophysiology of AD.

A previous study examining the correlation between [^18^F]THK-5351 PET and MRI revealed that regional tau deposition correlated with extrahippocampal subregional atrophy [6]. A similar finding was shown using a different radioactive tracer, AV-1451, with the AD cortical signature regions of medial, inferior, and lateral temporal lobes, as well as the inferior parietal lobule, exhibiting significant correlations with PET uptake [7]. Thus, tau PET binding in temporal and parietal regions may be useful for the staging of AD. However, studies demonstrating the relationship between tau PET binding and regional atrophy across the AD spectrum, including in mild cognitive impairment (MCI), are limited becasue the subject groups have generally been confined to AD patients and normal controls (NCs). Furthermore, the association may be modified by the β-amyloid status of patients, with increased β-amyloid in the cerebral spinal fluid (CSF) showing an association with hippocampal atrophy [7], although this has not been demonstrated using noninvasive imaging modalities.

[^18^F]THK-5351 PET is one of the first generation of noninvasive tau imaging agents; it shows a low binding affinity for white matter and a high binding affinity for tau aggregates [8]. In the present study, we compared THK5351 uptake over the entire cortical region with cortical atrophy measured on MRI, and we evaluated the relationship according to the subjects’ positions on the AD spectrum. Furthermore, we assessed the associations between cortical atrophy, THK-5351 PET uptake, and β-amyloid PET positivity in patients with AD and MCI, and NCs. The purpose of this study was to assess the *in vivo* whole-brain relationship between the uptake of [^18^F]THK-5351 on PET and cortical atrophy on structural MRI according to the presence and severity of AD.

## Materials and methods

### Participants

**Fig 1:**
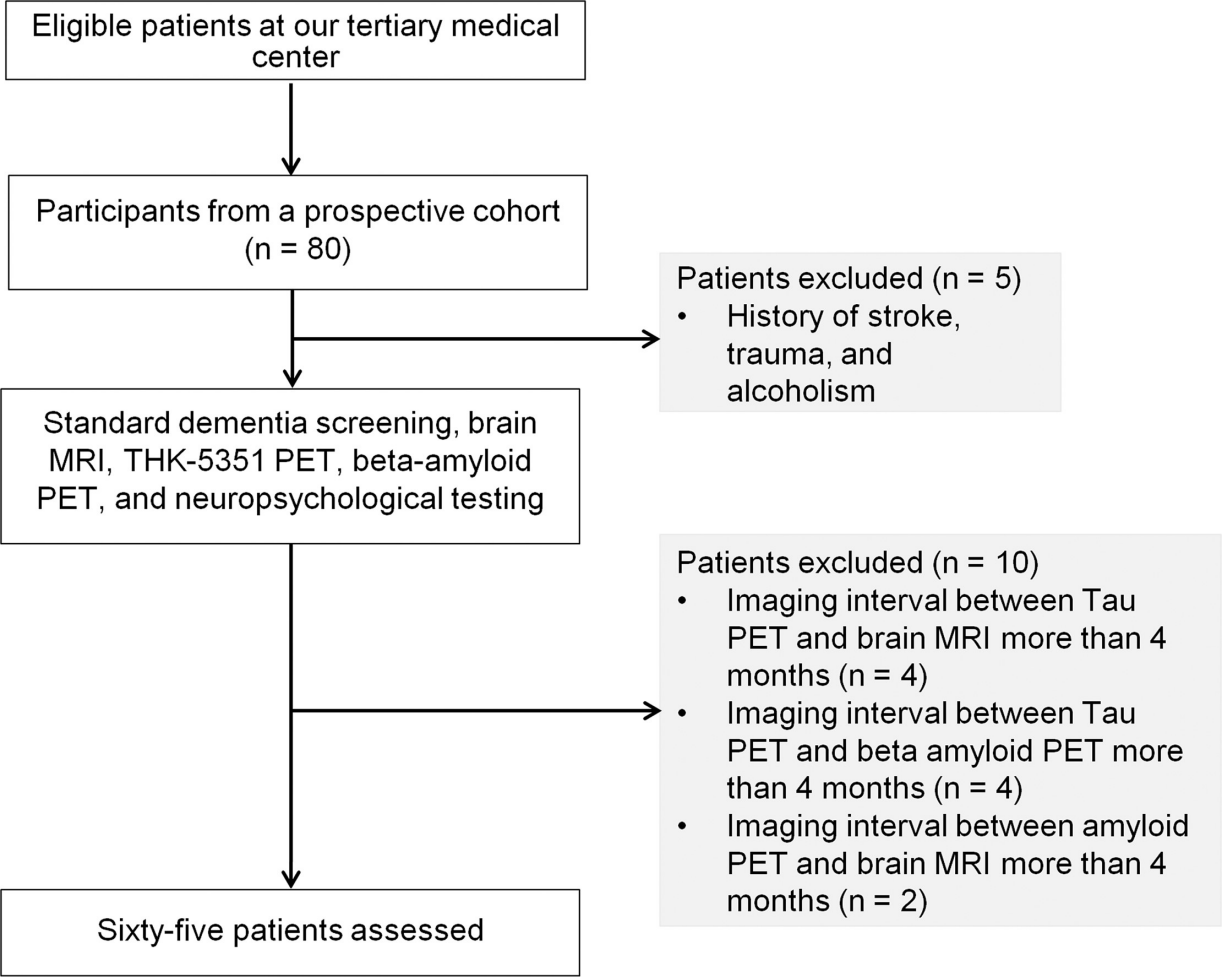
Flow diagram of the participant inclusion process of the study.

A flow diagram detailing the study participants is shown in **Fig 1**.

Data from 80 participants from a prospective cohort of a multicenter clinical trial (NCT02656498) were initially evaluated for inclusion in the present study. All participants provided informed consent and were examined under protocols approved by the institutional review board of the two tertiary medical centers. The participants had capacity to provide consent. If participants did not have capacity to provide consent, the consent was obtained from caregivers/guardians. All participants underwent standard dementia screening, including recording of medical history, physical examination, brain MRI, THK-5351 and amyloid PET, and neuropsychological testing. Clinical diagnosis was performed according to standard research criteria for probable AD [9], applied across a multidisciplinary team. Participants who were assigned as clinically normal (NCs) had a Mini-Mental State Examination score > 1.5 standard deviations above the normative mean and were without current clinical depression (Geriatric Depression Scale < 11). Participants were excluded if they had a history of stroke, head trauma, or alcoholism. Participants were further excluded when there was more than 160 days between the brain MRI, THK-5351, and amyloid PET, calculated from the first to last examination. The 65 finally enrolled participants included 21 NCs, 32 participants with MCI, and 12 AD patients. The demographic data of the participants are shown in **Table 1**. All relevant data are within the Supporting Information files.

**Table 1:** Demographics of the study participants.

	NC	MCI	AD	*P value*
N	21	32	12	
Age (years, median)	71 (IQR 66–75)	71.5 (IQR 67.5–76)	61 (IQR 55–69)	.011
Sex (M/F)	6/15	14/18	8/4	.104
Education (years, median)	9 (IQR 6–14.5)	12 (IQR 7.5–16)	9 (IQR 6–16)	.339
APOE (0/1/2 ε4 allele)	(17/3/0) (NA 1)	(15/12/1) (NA 4)	(8/1/1) (NA 2)	.395
MMSE score (median)	29 (IQR 27.8–29.2)	26 (IQR 23–27)	19 (IQR 15.7–21.7)	< .001
β-amyloid PET (+)	0	16 (50%)	9 (75%)	< .0001
Days between MRI and [^18^F]THK-5351 PET	56 (IQR 25–88)	41 (IQR 20.5–83)	51.5 (IQR 28–93)	.969
Days between MRI and beta-amyloid PET	23 (IQR 10–37)	21 (IQR 11–38.5)	9 (IQR 5–18.5)	.21
Days between [^18^F]THK-5351 PET and beta-amyloid PET	15 (IQR 3.7–63.7)	36 (IQR 1–70.2)	36.5 (IQR 15–67.5)	.70

Abbreviations-NC, normal controls; MCI, mild cognitive impairment; AD, Alzheimer’s disease; MMSE, Mini-Mental State Exam; IQR, interquartile range

### MRI acquisition

MRI was performed with a 3.0-T system (Achieva; Philips Medical Systems) using a sensitivity-encoding (SENSE), eight-channel head coil. A high-resolution anatomical three-dimensional (3D) volume image was obtained using a 3D gradient-echo T1-weighted sequence with the following parameters: repetition time/echo time, 9.9/4.6 ms; flip angle, 8°; field of view, 224 × 224 mm; matrix, 224 × 224; and slice thickness, 1 mm with no gap.

### [^18^F] THK-5351 PET imaging acquisition

All PET images were acquired using Discovery 690, 710, or 690 Elite PET/CT scanners (GE Healthcare;) at Asan Medical Center, or a Discovery STE PET/CT scanner (GE Healthcare) at Samsung Medical Center, with the same imaging and reconstruction protocols. [^18^F]THK-5351 PET images were obtained for 20 minutes, starting 50 minutes after injection of 185 ± 18.5 MBq of [^18^F]THK-5351. After the PET acquisition, the final image was obtained by summing all the individual images. The image quality of this representative image was checked for motion, image contrast, and noise by two nuclear medicine board certified physicians (M.Y.O. and J.S.K., with 6 and 20 years of experience, respectively). The PET images of the included participants were determined to be qualitatively adequate.

### β-amyloid PET acquisition and determination of β-amyloid positivity

β-amyloid PET imaging was performed on all participants using the aforementioned scanners. β-amyloid PET images were obtained for 20 minutes, starting 90 minutes after injection of 300 ± 30 MBq of [^18^F]florbetaben. PET images were assessed visually by the aforementioned nuclear medicine board certified physicians to determine regional cortical uptake in the frontal, lateral temporal, precuneus/posterior cingulate, and parietal regions. Patients were assigned as β-amyloid PET positive if they showed increased uptake in any of the four brain regions [10].

### Quantitative image analysis

[^18^F]THK-5351 PET and T1-weighted MR images were segmented using the default automated gyral-based parcellation method of FreeSurfer (version 5.3.0; http://surfer.nmr.mgh.harvard.edu) [11].

The standardized uptake values (SUVs) from the [^18^F]THK-5351 PET, and the cortical volumes (expressed as mm^3^) from the T1-weighted MR images, were extracted directly from the automated segmentation tool of FreeSurfer. The SUV ratio (SUVR) of the cerebral cortex was calculated using the SUV of the cerebellar cortex as a reference region, and cortical volume was normalized for head size using the total intracranial volume (also taken from FreeSurfer). Cortical volume was calculated to facilitate intra-regional correlations between the two imaging examinations. For each hemisphere, 34 surface-based regions were automatically defined using the Desikan-Killiany cortical atlas [12]. Further parcellations from the automatic FreeSurfer segmentation were utilized for the left and right hippocampus. The values for the left and right hemispheres were then averaged, and 35 regions were used in the analysis.

### *In vivo* Braak composite locations

The regions were expressed as locations in *in vivo* Braak staging [13], approximating the anatomical definitions of transentorhinal, limbic, and isocortical locations. These regions were previously demonstrated to be associated with neuropathologic staging on autopsy, which suggests that tau propagation starts at the medial temporal lobe and then spreads to the neocortex [1, 14]. The transentorhinal locations include entorhinal cortex and hippocampus. The limbic locations include fusiform, lingual, parahippocampal, inferior temporal, middle temporal, temporal pole, caudal anterior cingulate, isthmus cingulate, posterior cingulate, rostral anterior cingulate, and insula cortex. The isocortical locations include pars opercularis, pars orbitalis, pars triangularis, caudal middle frontal, lateral orbitofrontal, medial orbitofrontal, rostral middle frontal, superior frontal, frontal pole, bank of the superior temporal sulcus, superior temporal, transverse temporal, precuneus, supramarginal, inferior parietal, superior parietal, lateral occipital, cuneus, pericalcarine, paracentral, postcentral, and precentral cortex.

### Statistical analysis

The demographic characteristics represented by continuous variables and time intervals are expressed as median and interquartile range (IQR), while the THK-5351 uptake and cortical atrophy are expressed as mean and standard deviation. The characteristics of each group of participants (NC, MCI, and AD) were compared using chi-square tests for categorical variables and the Kruskal-Wallis test for continuous variables.

First, the Pearson correlation between THK-5351 uptake and cortical atrophy was estimated for each regions in the NCs and patients with AD spectrum disorders. The relationships were then compared among the NC, MCI, and AD groups, and a subgroup analysis was performed to investigate whether the relationships between SUVR and cortical atrophy were associated with β-amyloid PET positivity. A *P*-value < .05 was considered statistically significant.

The cortical volume and SUVR were then compared according to the disease group using an Analysis of Covariance (ANCOVA) with baseline age as a covariate. Post-hoc analysis between each group was further conducted. The exact *P*-values were calculated and statistical significance was set using a false discovery rate (FDR)-corrected *P*-value < .05 to account for multiple comparisons across the multiple brain locations. Both FDR-corrected and exact P-values are given.

All statistical analyses were performed using R statistical software (version 3.3.3, R Core Team).

## Results

### Participants

The participant characteristics are summarized in **Table 1**. In brief, the mean age was significantly lower in the AD patients than in the other groups. Gender ratio, education level, and APOE allele variations were similar between the groups. β-amyloid PET positivity was significantly different between the groups, with rates of 75% in the AD group and 50% in the MCI group, and with no subjects being β-amyloid PET positive in the NC group. As expected, AD patients performed significantly worse on the MMSE (*P* < .001) than the other groups. The mean intervals between [^18^F]THK-5351 PET and MRI, [^18^F]THK-5351 PET and β-amyloid PET, and β-amyloid PET and MRI were 43 days (median, interquartile range [IQR];19.5–88.2), 29 days (median, IQR; 1.5–58.5), and 22 days (median, IQR; 9.2–40.5), respectively. There was no significant difference in imaging intervals between the disease groups.

### Relationship between THK-5351 uptake and cortical volume according to AD spectrum

Intra-regional relationships between SUVR and cortical volume were calculated for the NC, MCI, and AD groups (**Table 2**).

**Table 2:** Comparison of locations showing correlations between THK-5351 uptake and cortical volume, demonstrated with *in vivo* Braak composite locations across the Alzheimer’s disease spectrum.

	Significant locations	NC (n = 21)	MCI (n = 32)	AD (n = 12)
Transentorhinal	Entorhinal	-0.132 (.561)	**-0.368** (.038)	**-0.697** (.012)
	Hippocampus	0.254 (.265)	-0.260 (.150)	-0.474 (.118)
Limbic	Parahippocampal	-0.119 (.607)	**-0.506** (.003)	-0.372 (.232)
	Middle temporal	-0.238 (.300)	**-0.442** (.011)	**-0.624** (.030)
	Temporal pole	-0.031 (.895)	**-0.571** (< .001)	-0.442 (.150)
	Isthmus cingulate	-0.216 (.346)	-0.073 (.689)	**-0.622** (.031)
Isocortical	Pars triangularis	-0.345 (.126)	**-0.371** (.037)	-0.306 (.333)
	Lateral orbitofrontal	0.018 (.938)	**-0.435** (.013)	-0.516 (.086)
	Superior frontal	-0.278 (.221)	-0.176 (.335)	**-0.614** (.033)
	Superior temporal	-0.121 (.600)	-0.259 (.152)	**-0.606** (.037)
	Precuneus	-0.107 (.543)	-0.284 (.230)	**-0.740** (.006)
	Supramarginal	-0.188 (.414)	-0.228 (.210)	**-0.576** (.049)
	Inferior parietal	-0.141 (.543)	-0.218 (.230)	**-0.781** (.003)
	Superior parietal	0.042 (.855)	-0.112 (.541)	**-0.683** (.014)

Note.-Correlation coefficients were calculated using Pearson’s correlation. Each location is represented by the average value of the left and right regions. The numbers in parentheses are *P*-values. Bold text indicates locations exhibiting statistical significance.

No location exhibited a significant relationship in the NC group. In the MCI group, six locations demonstrated a significant negative correlation between SUVR and cortical volume. These were the entorhinal (*r* = −0.368, *P* = .038), pars triangularis (*r* = −0.371, *P* = .037), lateral orbitofrontal (*r* = −0.435, *P* = .013), middle temporal (*r* = −0.442, *P* = .011), parahippocampal (*r* = −0.506, *P* = .003), and temporal pole (*r* = −0.571, *P* <. 001) regions, in reverse order of negative correlation strength. In the AD group, nine locations exhibited a significant negative correlation between SUVR and cortical volume, with these being the supramarginal (*r* = −0.576, *P* = .049), superior temporal (*r* = −0.606, *P* = .037), superior frontal (*r* = −0.614, *P* = .033), isthmus cingulate (*r* = −0.622, *P* = .031), middle temporal (*r* = −0.624, *P* = . 030), superior parietal (*r* = −0.683, *P* = . 014), entorhinal (*r* = −0.697, *P* = . 012), precuneus (*r* = −0.740, *P* = .006), and inferior parietal regions (*r* = −0.781, *P* = .003). More widespread and stronger correlations were observed in the AD group than in the other groups. The entorhinal and middle temporal cortex exhibited significant correlations in both the AD and MCI groups, although the relationship was stronger in the AD group (entorhinal, *r* = −0.697; middle temporal, *r* = −0.624) than in the MCI group (*r* = −0.368 and *r* = −0.442).

**Fig 2:**
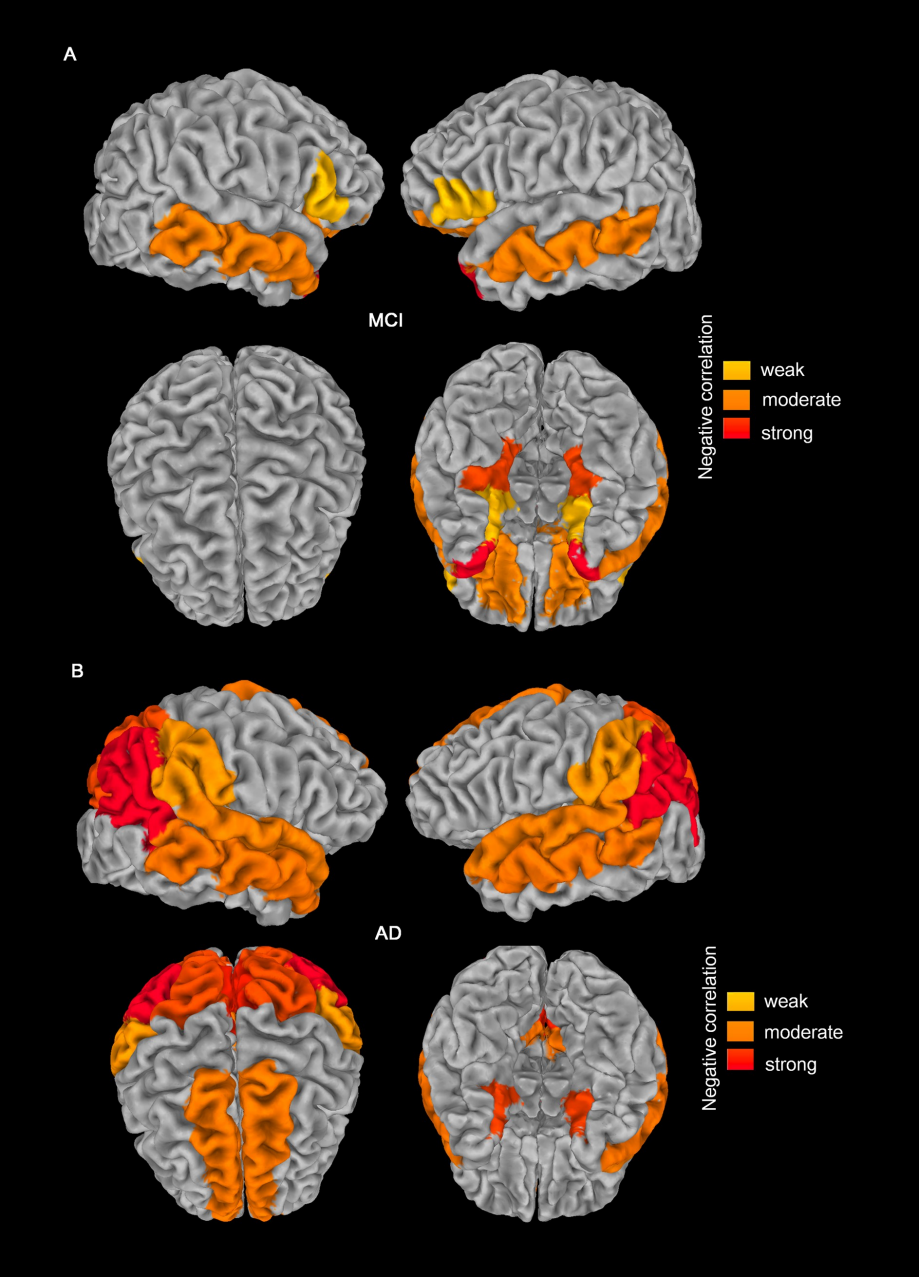
Regions exhibiting a significant negative relationship between THK5351 uptake and cortical atrophy in patients with mild cognitive impairment (MCI; A) and Alzheimer’s disease (AD; B). In MCI, the locations showing correlation occurred in one transentorhinal region (entorhinal), three limbic regions (parahippocampal, middle temporal, temporal), and two isocortical regions (pars triangularis and lateral orbitofrontal). In AD, correlated locations were found in one transentorhinal region (entorhinal), two limbic regions (middle temporal and isthmus cingulate), and six isocortical regions (superior frontal, superior temporal, precuneus, supramarginal, inferior parietal, and superior parietal). More widespread and stronger correlations were observed in the AD group.

**Fig 3:**
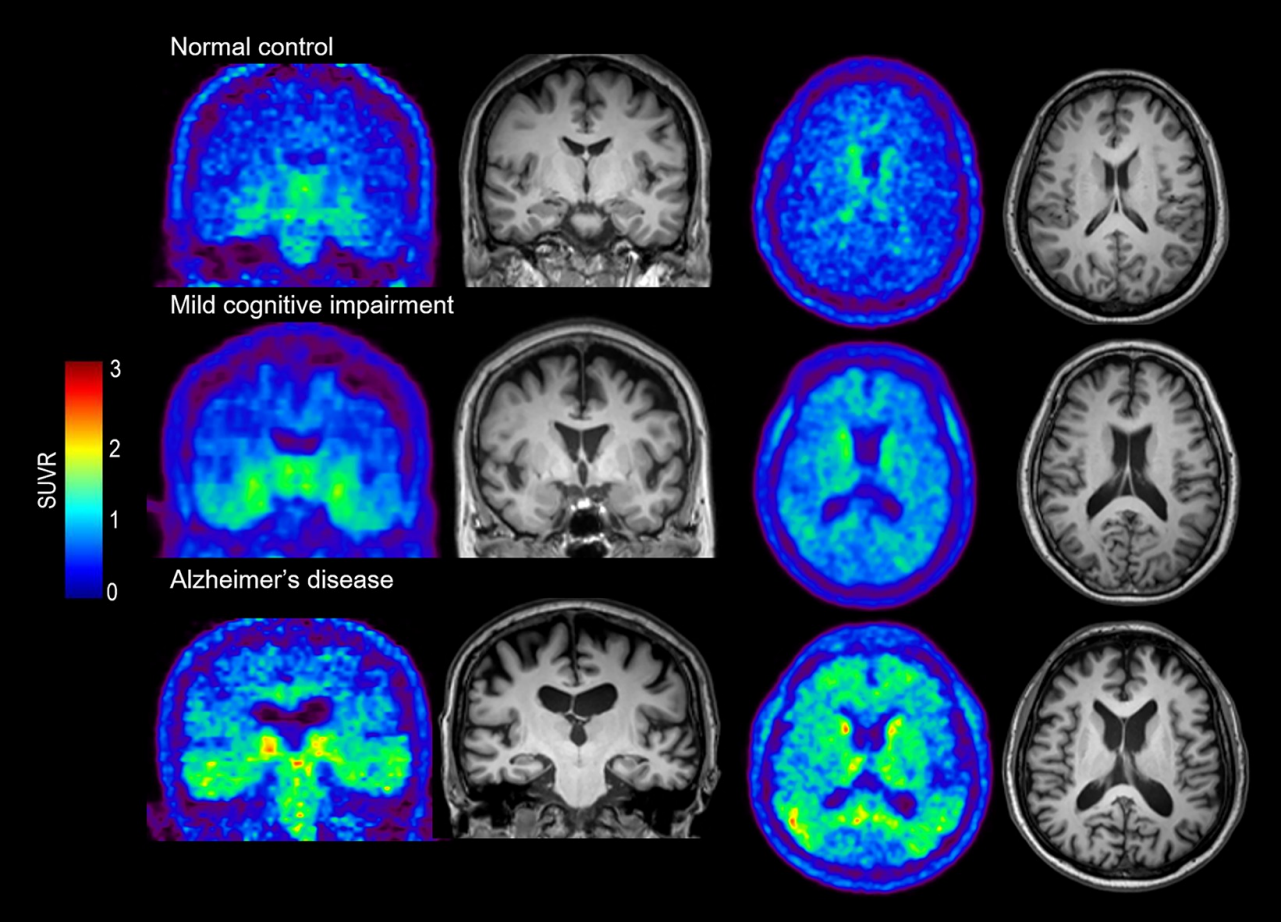
Representative cases of the relationship between THK5351 uptake and cortical atrophy in normal control, mild cognitive impairment, and Alzheimer’s disease. Note that the correlated locations propagate from transentorhinal to isocortical locations according to the AD spectrum.

When aligned with the *in vivo* Braak composite locations, the distribution of correlated locations differed between the MCI and AD groups, with more transentorhinal and limbic locations in MCI, but a broad extension to isocortical locations in AD, which is in accordance with the *in vivo* Braak staging based on tau propagation (**Fig 2**). Representative cases are shown in **Fig 3**.

### Effect of β-amyloid PET positivity on the relationship between THK-5351 uptake and cortical volume

**Table 3** summarizes the results comparing β-amyloid PET-positive and -negative groups over the entire study population.

**Table 3:** Comparisons between β-amyloid PET-positive and PET-negative groups.

	Significant locations	Aβ (+) (n = 40)	Aβ (-) (n = 25)
Transentorhinal	Entorhinal	**-0.602** (.001)	-0.221 (.170)
	Hippocampus	**-0.494** (.012)	-0.115 (.481)
Limbic	Parahippocampal	**-0.706** (< .001)	-0.267 (.095)
	Middle temporal	**-0.625** (.0008)	**-0.404** (.010)
	Temporal pole	**-0.422** (.036)	**-0.418** (.007)
	Isthmus cingulate	-0.364 (.073)	**-0.440** (.004)
Isocortical	Pars triangularis	**-0.524** (.037)	-0.112 (.491)
	Lateral orbitofrontal	**-0.436** (.029)	-0.112 (.490)
	Superior frontal	**-0.481** (.015)	-0.162 (.317)
	Superior temporal	**-0.483** (.014)	-0.121 (.457)
	Precuneus	-0.409 (.052)	**-0.515** (< .001)
	Supramarginal	**-0.409** (.042)	-0.308 (.053)
	Inferior parietal	**-0.459** (.021)	-0.346 (.263)
	Superior parietal	-0.224 (.282)	-0.351 (.052)

Note.-Correlation coefficients were calculated using Pearson’s correlation. Each location is represented by the average value of the left and right regions. The numbers in parentheses are *P*-values. Bold text indicates locations exhibiting statistical significance.

**Fig 4:**
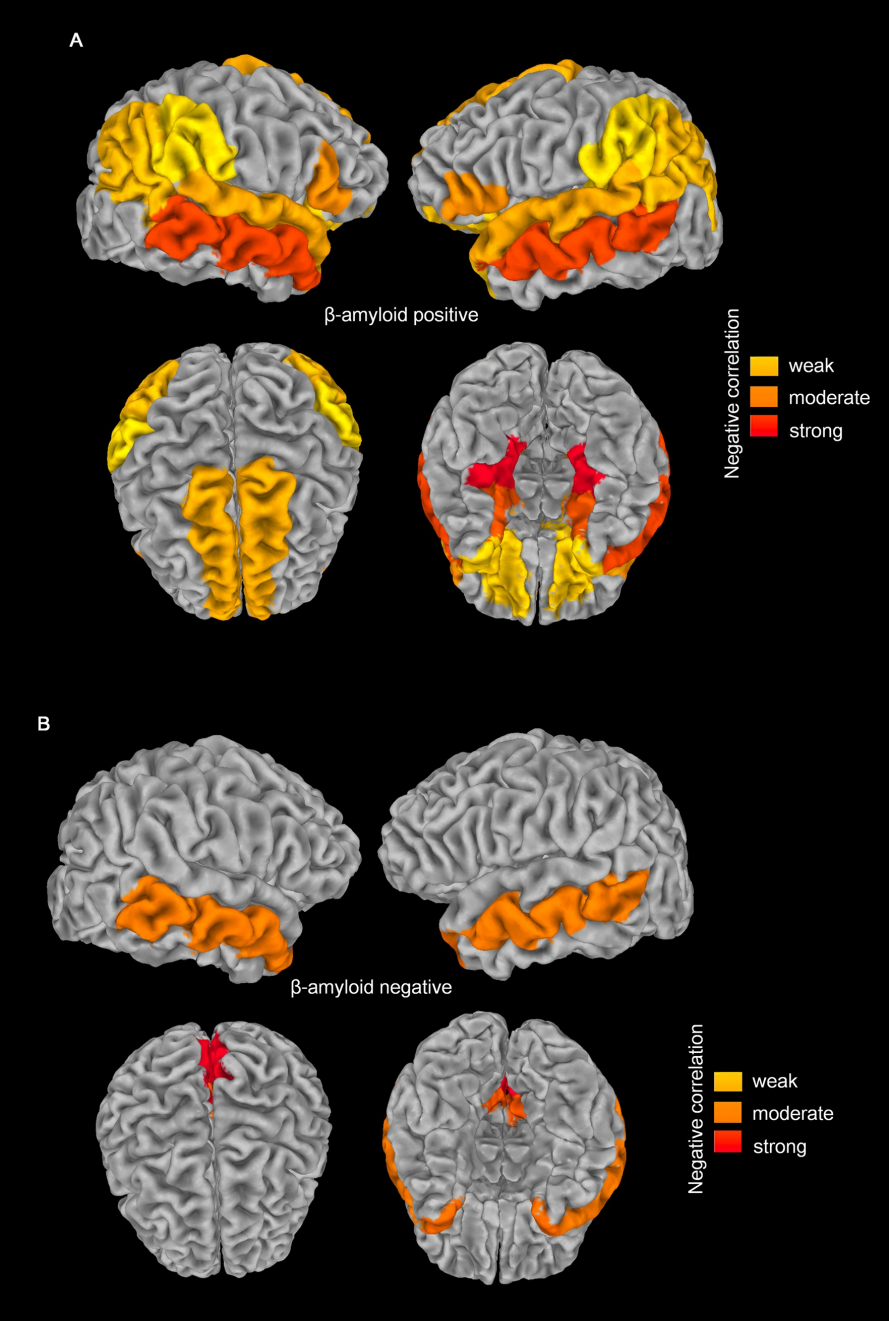
Differences in regions exhibiting significant negative relationships between THK5351 uptake and cortical atrophy in the β-amyloid positive (A) and -negative (B) groups. More widespread significant negative relationships were found in the β-amyloid positive group. Hippocampal atrophy was associated with THK5351 uptake only in the amyloid beta-positive group.

In the β-amyloid PET-negative group, middle temporal (*r* = −0.404), temporal pole (*r* = −0.418), isthmus cingulate (*r* = −0.440), and precuneus (*r* = −0.515) regions exhibited a mild negative relationship between THK5351 SUVR and cortical volume. The β-amyloid PET-positive group exhibited widespread and stronger correlations in multiple locations compared with the β-amyloid PET-negative group. In the β-amyloid PET-positive group, hippocampal formations exhibited associations between THK-5351 SUVR and cortical volume (*r* = −0.494, *P* = .012), which were not detected when β-amyloid PET was not considered. **Fig 4** demonstrates differences in the correlated locations between β-amyloid PET positive and negative groups.

Within the β-amyloid PET-positive group, the regional correlations were compared between the MCI and AD groups. **S1 Table** summarizes the result. There was trend for stronger correlations in AD group than in the MCI group in the entorhinal cortex (*r* = −0.770, *P* = .015) and superior frontal cortex (*r* = −0.688, *P* = .040).

### Locations exhibiting significant group difference according to the AD spectrum

**S2 Table** summarizes the results from locations showing significant group difference in SUVR and cortical volume.

Three limbic regions locations and five locations in isocortical regions showed significant differences in the THK-5351 SUVR in the disease group after age correction. Lingual, inferior temporal, pericalcarine, postcentral, and paracentral locations exhibited group differences, but these locations were different to those showing close correlations between THK-5351 PET and cortical volume. Furthermore, middle temporal, lateral orbitofrontal, and superior frontal locations exhibited both significant group-wise differences in association with disease severity, and close relationships in the correlation analysis between THK-5351 PET and cortical volume.

After age correction, three locations in the disease group that showed cortical volume differences on structural MRI showed significant differences in THK-5351 PET SUVR. Both parahippocampal cortex and hippocampus exhibited group differences, as well as close relationships between THK-5351 PET and cortical volume, particularly in the β-amyloid PET-positive group. In particular, the parahippocampal cortex exhibited the strongest correlation within the β-amyloid PET-positive group.

## Discussion

Our results show that, in patients on the AD spectrum, increased regional tau deposition measured with THK-5351 PET was significantly associated with cortical volume changes measured on structural MRI. Not only was the correlation strength between THK-5351 uptake and cortical volume stronger, but also the extent of the associated regions was greater, in the AD group than in the MCI group. The distribution of the correlated regions resembles that of *in vivo* Braak staging of tau propagation, with more isocortical distribution in AD than in MCI. Hippocampal atrophy did not show a relationship with THK-5351 deposition in the β-amyloid PET-negative group, but it did exhibit an association in the β-amyloid PET-positive group. Our noninvasive imaging results show a close relationship between the neuropathologic process and cortical atrophy on the AD spectrum, with a possible interaction between β-amyloid deposition and hippocampal atrophy.

This study presents three novel findings. First, the AD group showed stronger regional correlation between THK-5351 and cortical volume on MRI than did the MCI group. ^18[^F]AV-1451 PET imaging studies also revealed similar findings, in that significant local relationships were seen in temporal and parietal regions [15, 16]. This is supported by an autopsy study showing that the medial and lateral temporal lobes [5] exhibited high tau burden on pathology, as well as greater gray matter loss. However, the above studies did not include MCI patients, and the present study differs in that it showed that the distinct topographic relationship between THK-5351 uptake and cortical volume differed according to disease severity accross the AD spectrum. The MCI group showed mild to moderate negative correlations between THK-5351 uptake and cortical atrophy, while the AD group showed stronger correlations within the same locations, including entorhinal cortex (*r* = −0.368 in MCI and *r* = −0.697 in AD) and middle temporal cortex (*r* = −0.442 in MCI and *r* = −0.624 in AD). These results mirror the neuropathologic studies showing a close relationship between tau deposition, disease severity of AD, and neurodegeneration [17–19].

Second, hippocampal atrophy was significantly correlated with THK-5351 uptake in the β-amyloid PET positive group, but this correlation was not demonstrated in the β-amyloid PET-negative group. This may suggest that hippocampal atrophy is a result of an interaction between β-amyloid accumulation and the neurodegeneration process. A previous THK-5351 study supports this hypothesis, in that it found that regional tau deposition correlated with extrahippocampal subregional atrophy, not with hippocampal subfields [6]. The hippocampus has long been known to be a key region affected by atrophy in the early stage of AD [20–22], and the stronger correlation observed in extrahippocampal regions could be explained by the complex interplay of neurogeneration, taupathy and amyloid accumulation in the hippocampus [23, 24].

Third, the correlation between cortical atrophy and THK-5351 uptake is reflected in the Braak staging, which provides valuable information of propagation of neurodegeneration process. Neurofibrillary tangles initially occur in the entorhinal cortex, followed by involvement of the hippocampus, then progression to the temporal cortex and other cortical areas [1, 25]. An autopsy study also found that significant associations between neurofibrillary tangles and thinner cortical volume were strongest in the inferior temporal and parietal cortices, which correspond to Braak stages III and IV [5]. These locations were concordant with those in the present study, where the entorhinal cortex, precuneus, inferior parietal cortex, and superior parietal cortex showed the strongest associations in the AD group. Our results support the possible interaction of β-amyloid and tau pathology in hippocampal atrophy [23, 24], as well as atrophy in other parts of the temporal and parietal lobes.

The THK-5351 uptake can be confounded by monoamine oxidase B activity [26], which is expressed from reactive astrocytes because of neuroinflammation. However, a head-to-head comparison of THK-5351 and AV-1451 showed that THK-5351 mirrored neurodegeneration in a similar manner to AV-1451 [27], even though AV-1451 exhibited more specific tau-related information. A recent pathologic-imaging correlation study of [^18^F]THK5351 PET in postmortem patients [28] revealed that both Tau burden and astrogliosis affect [^18^F]THK5351 PET uptake in AD patients. Neuroinflammatory elements progress throughout the course of AD, with reactive astrocytes and activated microglia increasing as secondary processes followed by β-amyloid accumulation [29]. This shows potential of [^18^F]THK-5351 PET for monitoring the neuroinflammatory process in the living brain. Indeed, we found not only the amount of [^18^F]THK-5351 PET uptake, but also its correlation with cortical atrophy, was stronger in AD than in MCI, which supports the concept of using [^18^F]THK5351 PET uptake to demonstrate disease severity. Also, the study is strengthened by the facts that we performed correlations for all cortical locations, including the hippocampus, and separately analyzed patients according to AD spectrum and β-amyloid PET positivity.

There were several limitations to this study. First, we did not have pathological findings from autopsy to confirm the tau deposition. Second, the median interval between the THK-5351 and MRI scans was 47 days, between the amyloid PET and MRI was 29 days, and between the THK-5351 and amyloid PET was 22 days. The long delay between THK-5351 and MRI scans may have affected intra-individual within-region associations. Third, MCI and AD group included PIB-negative patients, and these cases may be limbic-predominant age-related TDP-43 encephalopathy or tauopathy other than AD (grain disease and tangle only dementia). Subgroup analysis of PIB-positive patients showed the same trend, but the statistical power is limited because of the small number of patients. A future study with larger cohort deemed to be necessary. Finally, the quantitative analysis was based on the same VOI and the vertexwise correlation analysis was not performed. Our observation serves to demonstrate the global relationship between THK-5351 uptake and cortical atrophy, indicating increased correlation between THK-5351 uptake and cortical atrophy in isocortical locations in AD compared with MCI or NC. Future studies using vertex-to-vertex correlation analysis may reveal spatial heterogeneity within VOIs.

In conclusion, regional THK-5351 uptake correlated more strongly with cortical atrophy in patients further along the AD spectrum, thereby demonstrating a close relationship between the neuropathologic process and cortical atrophy. Hippocampal atrophy was associated with both β-amyloid and THK-5351 uptake, possibly reflecting an interaction between β-amyloid and tau deposition in the neurodegeneration process.

## Supporting information

S1 Table Locations showing correlations between THK-5351 uptake and cortical volume, demonstrated with *in vivo* Braak composite locations across the Alzheimer’s disease spectrum in the amyloid PET-positive patients. (DOCX)

S2 Table Locations exhibiting significant group-wise difference between THK-5351 uptake and cortical atrophy with age correction. (DOCX)
