# Supplementary material for: Intra-individual correlations between quantitative THK-5351 PET and MRI-derived cortical volume in Alzheimer’s disease differ according to disease severity and amyloid positivity
Source: PLoS One. 2019 Dec 13;14(12):e0226265. doi: 10.1371/journal.pone.0226265 (PMC6910674; doi:10.1371/journal.pone.0226265)
Supplement: S1 Table — (DOCX) [file pone.0226265.s001.docx]

**Supplementary Table 1. Locations showing correlations between THK-5351 uptake and cortical volume, demonstrated with *in vivo* Braak composite locations across the Alzheimer’s disease spectrum in the amyloid PET-positive patients**

|  | Significant locations | **MCI**  **(n = 16)** | **AD**  **(n = 9)** |
| --- | --- | --- | --- |
| Transentorhinal | Entorhinal | -0.370 (.158) | **-0.770** (.015) |
|  | Hippocampus | -0.223 (.407) | -0.404 (.282) |
| Limbic | Parahippocampal | -0.346 (.189) | 0.018 (.964) |
|  | Middle temporal | -0.308 (.247) | -0.560 (.117) |
|  | Temporal pole | -0.363 (.167) | -0.371 (.325) |
|  | Isthmus cingulate | -0.299 (.260) | -0.458 (.215) |
| Isocortical | Pars triangularis | -0.273 (.307) | -0.152 (.697) |
|  | Lateral orbitofrontal | -0.479 (.060) | -0.336 (.377) |
|  | Superior frontal | -0.004 (.988) | -**0.688** (.040) |
|  | Superior temporal | -0.347 (.188) | -0.591 (.093) |
|  | Precuneus | -0.234 (.383) | -0.544 (.130) |
|  | Supramarginal | -0.176 (.515) | -0.02 (.963) |
|  | Inferior parietal | -0.254 (.343) | -0.527 (.145) |
|  | Superior parietal | 0.078 (.776) | -0.440 (.236) |

Note.-Correlation coefficients were calculated using Pearson’s correlation. Each location is represented by the average value of the left and right regions. The numbers in parentheses are *P*-values. Bold text indicates locations exhibiting statistical significance.
