# Supplementary material for: Intra-individual correlations between quantitative THK-5351 PET and MRI-derived cortical volume in Alzheimer’s disease differ according to disease severity and amyloid positivity
Source: PLoS One. 2019 Dec 13;14(12):e0226265. doi: 10.1371/journal.pone.0226265 (PMC6910674; doi:10.1371/journal.pone.0226265)
Supplement: S2 Table — (DOCX) [file pone.0226265.s002.docx]

**Supplementary Table 2. Locations exhibiting significant group-wise difference between THK-5351 uptake and cortical atrophy with age correction**

|  |  | | **NC** | **MCI** | **AD** | ***P*-value** (uncorrected) | ***P*-value** (corrected)***** | *Post-hoc analysis* | | |  |
| --- | --- | --- | --- | --- | --- | --- | --- | --- | --- | --- | --- |
|  | **Location** | |  |  |  |  |  | ***NC vs. MCI*** | ***NC***  ***vs. AD*** | ***MCI vs. AD*** |  |
| **SUVR (ratio)** | | |  |  |  |  |  |  |  |  |  |
| Limbic | | Lingual | 1.24 | 1.27 | 1.40 | <.001 | .003 | .51 | **<.001** | **.003** |  |
|  |  | Middle temporal | 1.31 | 1.43 | 1.72 | <.001 | <.001 | .061 | **<.001** | **<.001** |  |
|  | | Inferior temporal | 1.40 | 1.53 | 1.78 | <.001 | <.001 | .081 | **<.001** | **.005** |  |
| Isocortical | | Lateral orbitofrontal | 1.44 | 1.52 | 1.67 | <.001 | .002 | .087 | **<.001** | **.008** |  |
|  |  | Superior frontal | 1.16 | 1.25 | 1.41 | <.001 | <.001 | **.031** | **<.001** | **.004** |  |
|  |  | Pericalcarine | 1.24 | 1.32 | 1.44 | <.001 | <.001 | **.04** | **<.001** | **.009** |  |
|  | | Postcentral | 1.04 | 1.08 | 1.17 | <.001 | .003 | .199 | **<.001** | **.007** |  |
|  | | Paracentral | 1.17 | 1.22 | 1.37 | <.001 | <.001 | .21 | **<.001** | **<.001** |  |
| **Cortical volume (mL)** | | |  |  |  |  |  |  |  |  |  |
| Trans-entorhinal | | Hippocampus | 3.95 | 3.53 | 3.04 | .002 | .011 | **.024** | **<.001** | .054 |  |
| Limbic | | Parahippocampal | 1.94 | 1.75 | 1.59 | .001 | .012 | **.025** | **.002** | .215 |  |
| Isocortical | | Bankssts | 2.22 | 2.03 | 1.81 | .001 | .013 | .075 | ***.*002** | .132 |  |

Note.-Age was adjusted using linear regression. **P*-values were corrected using false discovery rate. Bankssts: bank of superior temporal sulcus.
